# Artificial intelligence in African malaria control programs: opportunities and risks

**DOI:** 10.1097/MS9.0000000000004259

**Published:** 2025-10-31

**Authors:** Emmanuel Ifeanyi Obeagu, Fabian Chukwudi Ogenyi

**Affiliations:** a Department of Biomedical and Laboratory Science, Africa University, Mutare, Zimbabwe; b Department of Electrical, Telecommunication and Computer Engineering, Kampala International University, Ishaka, Uganda

**Keywords:** Africa south of the Sahara, algorithms, artificial intelligence, malaria/prevention & control, Plasmodium

## Abstract

Malaria remains a major public health challenge in Sub-Saharan Africa, contributing to significant morbidity and mortality despite decades of control efforts. Conventional interventions – including vector control, chemoprevention, and diagnostics – face limitations due to drug and insecticide resistance, climate variability, and health system constraints. Artificial intelligence (AI) offers transformative potential to strengthen malaria control programs by enhancing surveillance, predicting outbreaks, improving diagnostics, optimizing treatment, and guiding resource allocation. This narrative review synthesizes current applications of AI in African malaria programs, highlighting case studies such as Kenya’s drone-assisted vector surveillance, Nigeria’s mobile health platforms for real-time case reporting, and Tanzania’s climate-informed forecasting models. We further discuss AI-driven bioinformatics for *Plasmodium* genomic surveillance, modeling of parasite life cycle dynamics under environmental stress, and network-based analyses of vector–parasite molecular interactions. Challenges, including data quality, ethical considerations, infrastructure limitations, and potential inequities, are critically examined. By integrating practical examples with emerging AI methodologies, this review underscores both the opportunities and risks of AI in malaria control and provides guidance for policymakers, researchers, and public health practitioners aiming to leverage AI to accelerate malaria elimination in Africa.

## Introduction

Malaria remains one of the most pressing public health challenges in Africa, accounting for a significant proportion of global morbidity and mortality associated with the disease^[1]^. Despite considerable progress over the past two decades through interventions such as insecticide-treated nets, indoor residual spraying (IRS), and improved case management, malaria continues to impose a heavy burden on African health systems and economies^[2]^. The complex and dynamic nature of malaria transmission, influenced by factors such as climate variability, population movement, and parasite resistance, demands innovative and adaptive strategies to accelerate control and eventual elimination^[3]^. In this context, artificial intelligence (AI) has emerged as a transformative technology with the potential to revolutionize malaria control programs^[4]^. AI refers to a broad set of computational methods, including machine learning, deep learning, and natural language processing, that enable machines to mimic human intelligence by learning from data, identifying patterns, and making predictions^[5]^. These capabilities are particularly valuable in malaria control, where vast and heterogeneous datasets – ranging from epidemiological surveillance, environmental monitoring, and genomic data – require sophisticated analysis to extract actionable insights^[6]^.

AI-driven tools have demonstrated promising applications in several aspects of malaria control in Africa. For instance, machine learning algorithms have been used to analyze climatic and satellite data to predict malaria outbreaks and identify high-risk transmission hotspots^[7]^. Computer vision techniques have automated the detection of malaria parasites in blood smears, improving diagnostic accuracy and reducing reliance on overburdened laboratory personnel^[8]^. Furthermore, AI-powered mobile health (mHealth) applications support health workers in remote areas by providing decision support and facilitating real-time data reporting. Ethical considerations also loom large in the adoption of AI for malaria control. Issues related to data privacy, informed consent, and potential misuse of sensitive health information must be addressed through transparent governance frameworks^[9]^. Additionally, AI models trained on biased or unrepresentative data risk perpetuating health disparities by delivering inaccurate or inequitable outcomes^[10]^. This underscores the need for local involvement in the development, validation, and continuous monitoring of AI tools to ensure cultural relevance and fairness^[11]^.

Capacity building represents another critical factor for successful AI integration. Many healthcare providers and policymakers in Africa may lack the technical expertise required to understand, interpret, and effectively utilize AI-generated insights^[12]^. Bridging this skills gap through targeted training and education programs is essential to foster trust and empower local actors to actively participate in AI-driven malaria control initiatives^[13,14]^. In light of the evolving malaria landscape and the rapid advancement of AI technologies, this review aims to explore the opportunities and risks associated with the use of AI in African malaria control programs. By critically examining current applications, challenges, and future directions, we seek to inform policymakers, researchers, and practitioners on how best to leverage AI to enhance malaria surveillance, diagnosis, treatment, and ultimately contribute to sustainable malaria elimination on the continent.

## Aim

This review aims to critically assess the role of AI in malaria control programs across Africa by exploring current applications, identifying key opportunities for enhancing disease surveillance, diagnosis, and treatment, and examining the associated risks and challenges. The goal is to provide insights that can guide the ethical, equitable, and effective integration of AI technologies to accelerate malaria control and elimination efforts on the continent.

## Methods

This narrative review was conducted through a structured literature search to synthesize current knowledge on the opportunities and risks of applying AI in African malaria control programs. Relevant peer-reviewed articles, reports, and policy documents were identified by searching PubMed, Scopus, and Web of Science databases for publications between 2010 and 2025. The primary search terms included combinations of “Artificial Intelligence,” “Machine Learning,” “Malaria,” “Plasmodium,” “Africa,” and “Vector Control.” Additional records were retrieved from the World Health Organization malaria database and reference lists of key articles. English-language publications focusing on AI applications in malaria surveillance, diagnostics, treatment optimization, vector management, or policy implementation in African settings were considered. Evidence was critically appraised and narratively synthesized to highlight thematic opportunities, risks, and implementation considerations, without performing a formal meta-analysis.

### AI applications in malaria surveillance and diagnosis

AI has shown considerable promise in enhancing malaria surveillance and diagnostic processes in Africa by enabling more accurate, timely, and scalable interventions^[9]^. In surveillance, AI techniques – particularly machine learning algorithms – are leveraged to analyze complex datasets that include climate variables, land use patterns, human mobility, and historical case reports^[15]^. By integrating these diverse data streams, AI models can identify transmission hotspots and predict outbreak risks with greater precision than traditional statistical methods^[16]^. For example, predictive analytics using satellite imagery and weather data have been used to forecast malaria incidence weeks in advance, allowing public health officials to allocate resources more efficiently and implement targeted vector control measures before outbreaks occur^[17]^.

In the realm of diagnosis, AI-driven tools are revolutionizing how malaria infections are detected and confirmed, especially in resource-limited settings where skilled microscopists may be scarce^[18]^. Computer vision and deep learning algorithms have been developed to automate the analysis of blood smear images, identifying malaria parasites with high accuracy and consistency while reducing human error and workload^[19]^. These AI-powered systems can rapidly screen large numbers of samples, enabling faster diagnosis and treatment initiation. Moreover, smartphone-based diagnostic applications utilize AI to analyze images of blood smears or rapid diagnostic test (RDT) results, bringing malaria diagnosis closer to remote communities and reducing reliance on centralized laboratory facilities^[20]^.

**HIGHLIGHTS**

- AI enhances malaria surveillance, outbreak prediction, and treatment optimization in Africa.
- Drone and mobile platforms improve vector mapping and real-time case reporting.
- Genomic AI detects resistance mutations in *Plasmodium*.
- Climate-informed models forecast transmission under environmental stress.
- Ethical, infrastructural, and equity challenges require careful management.

Beyond traditional microscopy, AI is also enhancing molecular diagnostic methods by assisting in the interpretation of complex data generated from polymerase chain reaction and loop-mediated isothermal amplification tests^[21]^. Machine learning models can help detect low-level parasitemia and differentiate between Plasmodium species, which is critical for appropriate treatment decisions and surveillance of drug resistance^[22]^. Additionally, AI tools facilitate real-time data aggregation and visualization, linking diagnostic results to health information systems to improve outbreak detection and response^[23]^. Together, these AI applications have the potential to transform malaria surveillance and diagnosis in Africa by increasing accuracy, accessibility, and speed, ultimately contributing to more effective malaria control and elimination strategies (Table 1).

**Table 1:** AI applications in African malaria control programs based

Country	AI application	Description	Measured/reported outcomes
Kenya	Drone-assisted vector surveillance	Drones capture high-resolution images of mosquito breeding habitats; AI image recognition identifies larval sites for targeted control	25–30% improvement in breeding site detection compared with traditional surveys
Nigeria	Mobile health AI platforms	Smartphone-based reporting and predictive analytics enable real-time malaria case tracking and hotspot detection	Reporting delays reduced from weeks to <48 hours; improved resource allocation for RDTs and antimalarials
Tanzania	AI-based climate–malaria forecasting	Machine learning models integrate satellite climate data (rainfall, temperature, humidity) to predict district-level malaria incidence	Early seasonal risk maps generated 1–2 months in advance closely match observed case trends; supports proactive spraying and interventions
Ethiopia	Machine learning for outbreak prediction	Integration of epidemiological and climate data to forecast malaria transmission trends	Improved early warning capacity; enabled pre-positioning of medical supplies in high-risk districts
Ghana	AI-assisted diagnostic image analysis	Deep learning applied to microscope and RDT images to improve detection of *Plasmodium* species	Sensitivity and specificity comparable to expert microscopists; reduced diagnostic errors in low-resource clinics

### *AI-driven bioinformatics for* Plasmodium *genomic surveillance*

AI is increasingly transforming malaria genomics by enabling the rapid and accurate analysis of complex *Plasmodium* datasets generated through next-generation sequencing. Traditional bioinformatics pipelines, while powerful, often struggle to handle the massive scale and heterogeneity of genomic data from field isolates. Deep learning algorithms, including convolutional neural networks and recurrent neural networks, are now being integrated into genomic workflows to overcome these challenges. These AI-driven tools excel at detecting subtle sequence patterns, single-nucleotide polymorphisms, and structural variants that may signal emerging drug resistance^[17]^. A prominent example is the identification of mutations in the kelch13 gene of *Plasmodium falciparum*, which is strongly associated with artemisinin resistance. AI models trained on large genomic datasets can recognize resistance-linked variants more efficiently than conventional approaches, even when these variants occur at low frequencies in diverse populations. By combining genomic signals with geospatial and epidemiological data, machine learning systems can also predict the potential spread of resistant strains across regions, providing early warnings to guide treatment policy and containment strategies^[18]^.

Beyond resistance detection, AI-driven bioinformatics facilitates phylogenetic analyses, tracks parasite population dynamics, and models evolutionary pressures under changing environmental conditions or drug regimens. These capabilities are particularly valuable for Africa, where the rapid detection and containment of resistant *Plasmodium* lineages are critical for preserving the efficacy of artemisinin-based combination therapies. Integrating AI-powered genomic surveillance into national malaria control programs can therefore strengthen decision making and support proactive interventions aimed at maintaining therapeutic effectiveness and advancing elimination goals (Table 2) ^[19]^.

**Table 2:** AI applications in Plasmodium genomics, parasite life cycle modeling, and vector–parasite molecular interactions

Application area	AI approach	Description	Key insights/outcomes
Genomic surveillance	Deep learning on next-generation sequencing data	AI analyzes *Plasmodium* genomic datasets to detect resistance mutations and predict the spread of resistant strains	Early identification of *kelch13* mutations associated with artemisinin resistance; prediction of high-risk regions for resistant parasite expansion
Life cycle modeling	Machine learning and reinforcement learning	AI integrates climate, drug pressure, and ecological data to simulate alterations in *Plasmodium* development and transmission	Forecasts how environmental stress and intermittent drug exposure may affect gametocyte maturation, parasite survival, and transmission intensity
Vector–parasite molecular interactions	Network analysis and molecular docking	AI identifies novel protein–protein interactions between parasite ligands and mosquito receptors; predicts binding affinities	Discovery of potential molecular targets for transmission-blocking vaccines or inhibitors; guides rational design of vector control interventions
Drug resistance prediction	Predictive modeling using genomic and pharmacological data	AI integrates mutation profiles with epidemiological and treatment data to forecast emergence of drug-resistant *Plasmodium* strains	Supports policy decisions on artemisinin-based combination therapy (ACT) deployment and containment strategies
Environmental risk forecasting	Climate-informed ML models	Combines satellite-derived environmental data with parasite biology to predict seasonal transmission risk and outbreak likelihood	Enables proactive vector control and resource allocation; informs timing of spraying and chemoprevention campaigns

### *AI-based ecological and evolutionary modeling of* Plasmodium *life cycle dynamics*

Recent advances in AI have enabled the development of ecological and evolutionary models that simulate how climate variability and drug pressure can influence the life cycle dynamics of *Plasmodium* parasites. These models integrate high-resolution environmental datasets – such as temperature, rainfall, humidity, and vegetation indices – with parasite biology to predict how shifting ecological conditions may affect the timing and intensity of transmission. Machine learning algorithms, including random forests and deep neural networks, can detect complex, nonlinear relationships between climate drivers and parasite developmental stages, offering more nuanced predictions than traditional statistical approaches^[20]^. Some experimental AI platforms couple climate data with genomic and pharmacological inputs to explore how selective drug pressure interacts with environmental stressors to shape parasite evolution. For example, reinforcement learning frameworks have been used to simulate how intermittent drug exposure or fluctuating vector populations might favor resistant genotypes, alter gametocyte maturation rates, or extend parasite survival within mosquito hosts. These models provide valuable foresight into how drug-resistant *Plasmodium* lineages could expand or decline under projected climate change scenarios^[21]^.

Although still in early stages, such AI-based ecological and evolutionary simulations represent a powerful tool for anticipatory malaria control. By forecasting changes in parasite development, transmission windows, and resistance emergence, these models can inform adaptive strategies – such as adjusting the timing of IRS, optimizing chemoprevention schedules, or prioritizing genomic surveillance in high-risk areas – thereby strengthening Africa’s preparedness for environmental and pharmacological challenges^[22]^.

### AI-driven network analysis of vector–parasite molecular interactions

AI is emerging as a key enabler of systems-level understanding of the molecular dialogue between *Plasmodium* parasites and their *Anopheles* mosquito vectors. The complex interplay of parasite proteins, mosquito midgut receptors, and immune factors determines the success of parasite development within the vector and ultimately drives transmission efficiency. AI-driven network analysis integrates multi-omics datasets – including proteomics, transcriptomics, and metabolomics – to identify previously unrecognized protein–protein interactions that facilitate parasite invasion, immune evasion, or gametocyte maturation^[23]^.

Machine learning algorithms can mine vast biological networks to detect critical interaction hubs and predict functional relationships that may not be apparent through conventional experimental methods. Complementing this, AI-powered molecular docking tools simulate three-dimensional binding between candidate parasite ligands and mosquito receptors, rapidly screening thousands of potential complexes to pinpoint high-affinity interactions. By revealing novel binding sites or structural motifs that are essential for parasite survival within the vector, these approaches can uncover promising targets for transmission-blocking interventions such as vaccines, small-molecule inhibitors, or gene-drive strategies^[24]^. Integrating these computational discoveries with laboratory validation accelerates the identification of vector–parasite contact points that can be disrupted to interrupt the malaria life cycle. For African malaria control programs, AI-enhanced molecular insights hold particular promise for the rational design of next-generation tools aimed at reducing vector competence and advancing toward sustainable malaria elimination^[25]^.

### Opportunities for optimizing treatment and resource allocation

AI offers significant opportunities to optimize malaria treatment strategies and improve the allocation of limited healthcare resources in Africa^[24]^. One of the key benefits of AI lies in its ability to analyze large and diverse datasets – such as patient clinical records, drug resistance patterns, and treatment outcomes – to identify the most effective therapeutic regimens tailored to specific populations or regions^[25]^. Machine learning models can assist clinicians in making personalized treatment decisions by predicting patient responses to antimalarial drugs, thereby reducing the risk of treatment failure and contributing to better health outcomes^[26]^. Furthermore, AI-driven predictive analytics support health system managers in optimizing resource allocation by forecasting demand for antimalarial medications, diagnostic supplies, and healthcare workforce needs^[27]^. These predictions enable proactive planning to avoid stockouts and ensure that essential commodities reach high-risk areas in a timely manner. In regions where malaria transmission is seasonal or influenced by environmental factors, AI can help adjust intervention schedules dynamically, such as timing IRS campaigns or mass drug administration programs for maximum impact^[28]^.

Another promising area is the use of AI-powered mHealth platforms that facilitate real-time data collection and communication between frontline health workers and central malaria control units^[12]^. These platforms can flag potential treatment complications, monitor adherence to drug regimens, and guide referral decisions, thereby improving case management at the community level^[29]^. By integrating AI into decision support systems, malaria control programs can enhance efficiency, reduce costs, and better target interventions to vulnerable populations, accelerating progress toward malaria elimination in Africa.

### Risks and challenges in AI integration

While AI offers transformative potential for malaria control in Africa, its integration into public health programs is accompanied by significant risks and challenges that must be carefully managed^[30]^.

1. **Data quality and bias**

AI systems rely on large, high-quality datasets for training and validation. In many African contexts, health information systems are fragmented, and case reporting may be incomplete or inconsistent. Inaccurate or biased input data can lead to erroneous predictions, misallocation of resources, and reinforcement of existing health disparities. Ensuring rigorous data cleaning, standardization, and representation across populations is therefore critical^[31,32]^.

2. **Privacy and ethical concerns**

Malaria surveillance increasingly involves geospatial, genomic, and personal health data. Without robust governance, AI applications risk violating privacy, consent, and data sovereignty. Ethical frameworks must address informed consent, secure data storage, controlled access, and equitable sharing of benefits arising from AI-driven analyses^[33,34]^.

3. **Infrastructure and capacity limitations**

AI deployment requires reliable electricity, internet connectivity, computational resources, and trained personnel. Many rural and underserved regions may lack these prerequisites, hindering adoption and sustainability. Investment in local infrastructure, digital literacy, and capacity building is essential to prevent dependence on external technology partners^[35,36]^.

4. **Algorithmic transparency and accountability**

Complex AI models, especially deep learning systems, often function as “black boxes,” making it difficult for policymakers or health workers to interpret predictions. Lack of transparency can erode trust, delay decision making, and complicate error correction. Explainable AI approaches and stakeholder training are necessary to enhance interpretability and account ability^[37,38]^.

5. **Inequality and technological dependency**

Unequal access to AI tools can exacerbate health inequities, favoring urban or well-resourced areas while leaving marginalized populations behind. Additionally, overreliance on externally developed AI solutions may undermine local ownership and capacity. Prioritizing open-source platforms, local innovation, and inclusive implementation strategies can mitigate these risks^[39,40]^.

6. **Sustainability and cost considerations**

Maintaining AI systems – including software updates, data management, and model retraining – requires ongoing financial and human resources. Without long-term planning, AI initiatives may become unsustainable once initial project funding ends. Integrating AI into existing national malaria control frameworks and securing multi-year support is essential for durable impact^[12,14,41,42]^.

### Case studies of AI applications in African malaria control

Several pioneering initiatives across Africa demonstrate how AI is being translated into practical tools for malaria surveillance, prediction, and control. These real-world examples highlight the diversity of AI applications and provide valuable lessons for scaling similar approaches across the continent.

### *Kenya* – *drone-assisted AI vector surveillance*

In western Kenya, national malaria control programs have piloted the use of unmanned aerial vehicles (drones) coupled with AI-driven image recognition to map mosquito breeding habitats with unprecedented precision. High-resolution aerial imagery is processed by machine learning algorithms to identify water bodies, vegetation density, and land-use patterns that support *Anopheles* larval development. Pilot results show a 25–30% improvement in breeding site detection compared with traditional ground surveys, enabling more targeted larviciding and optimized deployment of vector control resources^[43]^.

### *Nigeria* – *mHealth AI platforms for real-time case reporting*

Nigeria has implemented AI-enhanced mHealth platforms that integrate smartphone-based reporting, geolocation, and predictive analytics for near-real-time malaria case tracking. Health workers upload diagnostic results through mobile applications, where AI algorithms detect anomalies and predict emerging hotspots. Early assessments indicate a significant reduction in reporting delays – from weeks to less than 48 hours – facilitating faster distribution of RDTs and antimalarial treatments to high-burden areas^[44]^.

### *Tanzania* – *AI-based climate–malaria forecasting models*

In Tanzania, machine learning models using satellite-derived climate variables such as rainfall, temperature, and humidity are being tested to forecast malaria incidence at the district level. These AI-based systems generate seasonal transmission risk maps that guide the timing of IRS and community education campaigns. Preliminary evaluations show that predictions made 1–2 months in advance align closely with observed case trends, supporting proactive resource allocation^[45]^.

### Ethical considerations in AI-driven malaria control

The integration of AI into malaria control programs raises important medical and public health ethics considerations that extend beyond traditional concerns. AI applications increasingly involve sensitive data, including geolocation of cases, genomic information of *Plasmodium* parasites, and personal health records of affected populations. Ensuring informed consent, confidentiality, and secure data storage is essential to maintain trust between communities and health authorities^[46]^. Equitable access to AI-driven interventions is another critical concern. Without deliberate planning, AI tools may preferentially benefit urban or better-resourced areas, exacerbating existing health disparities. Strategies such as open-source platforms, inclusive training, and community engagement can help mitigate inequities and promote fair distribution of technological benefits.

Transparency and accountability in AI decision making are also ethical imperatives. Complex “black-box” models must be accompanied by explainable AI approaches to allow health workers and policymakers to interpret recommendations, verify accuracy, and make informed decisions. Additionally, cross-border data sharing for genomic surveillance or outbreak prediction must respect sovereignty and international ethical standards, ensuring that benefits derived from AI analytics are shared with the communities providing the data^[47]^. By proactively addressing privacy, equity, and transparency, AI can be deployed responsibly in African malaria programs. Ethical frameworks that integrate local values, legal standards, and community participation are critical for safeguarding rights while maximizing the public health impact of AI-driven malaria control.

### Future directions and recommendations

The future integration of AI in African malaria control programs offers promising avenues to accelerate disease reduction, but realizing this potential requires strategic planning and collaborative efforts^[48]^. First, expanding investments in digital infrastructure across malaria-endemic regions is essential to support reliable data collection, storage, and processing^[49]^. Improving internet connectivity, electricity access, and computing capacity will enable the deployment of sophisticated AI tools in even the most remote and resource-limited settings^[50]^. Capacity building is another cornerstone for sustainable AI adoption. Training healthcare workers, data scientists, and policymakers in AI literacy will empower local stakeholders to understand, interpret, and manage AI applications effectively^[51]^. Encouraging interdisciplinary partnerships between local universities, research institutions, and technology developers can foster homegrown innovations tailored to the unique epidemiological and socio-cultural context of African communities^[52]^.

To address ethical and governance challenges, establishing clear regulatory frameworks for data privacy, consent, and transparency is imperative^[53]^. AI models should be designed with explainability in mind, allowing users to comprehend how decisions are made and ensuring accountability^[54]^. Engaging communities throughout the AI development and implementation process will help build trust and ensure that interventions respect local values and priorities^[55]^. Furthermore, enhancing data quality through standardization and integration of health information systems is critical for building robust AI models^[56]^. Collaborative efforts between governments, international organizations, and private sector partners can promote the creation of interoperable platforms that facilitate real-time data sharing while safeguarding privacy^[57]^. Fostering innovation through pilot projects and operational research will provide valuable insights into the effectiveness, scalability, and cost-efficiency of AI tools in real-world malaria control settings^[58]^. Continuous monitoring and evaluation should be embedded into AI initiatives to identify potential unintended consequences and inform adaptive strategies^[59,60]^.

## Conclusion

AI presents transformative opportunities to enhance malaria control programs in Africa by improving surveillance accuracy, accelerating diagnosis, optimizing treatment, and enabling efficient resource allocation. However, realizing these benefits requires overcoming significant challenges related to data quality, infrastructure limitations, ethical concerns, and workforce capacity. Through targeted investments in digital infrastructure, capacity building, transparent governance, and community engagement, AI can be responsibly integrated into malaria control strategies. With collaborative and context-sensitive approaches, AI has the potential to accelerate progress toward malaria elimination and improve health outcomes across the continent.

## Data Availability

Not applicable as this is a narrative review.

## Research registration unique identifying number (UIN)

Not applicable as this is a narrative review.
